# Condom use and HIV testing among men who have sex with men in Jordan

**DOI:** 10.7448/IAS.17.1.18573

**Published:** 2014-04-01

**Authors:** Abdulsalam Alkaiyat, Christian Schaetti, Mohammad Liswi, Mitchell G Weiss

**Affiliations:** 1 Department of Epidemiology and Public Health, Swiss Tropical and Public Health Institute, Basel, Switzerland; 2 University of Basel, Basel, Switzerland; 3 Department of Community Medicine, Public Health and Family Medicine, Faculty of Medicine, Jordan University of Science and Technology, Irbid, Jordan

**Keywords:** HIV/AIDS, MSM, condom, HIV test, Jordan, Middle East and North Africa

## Abstract

**Introduction:**

To identify sociocultural determinants of self-reported condom use and HIV testing and examine variables related to accessibility, motivation and obstacles among men who have sex with men (MSM) in Jordan.

**Design:**

Cross-sectional study among MSM who were identified through services of a local non-governmental organization (NGO).

**Methods:**

Respondents were studied with a semi-structured interview based on the Explanatory Model Interview Catalogue (EMIC) framework. The vignette-based EMIC interview considered locally relevant HIV/AIDS-related knowledge, risk perception and perceived causes, as well as awareness of services and sources of support.

**Results:**

Of the 97 respondents, 27% reported that they used a condom at last intercourse; 38% had been tested at least once for HIV. Positive determinants of condom use were higher education level, acknowledging MSM as a high-risk group, seeking advice from a medical doctor and the perceived causes “sex with prostitutes” and “sex with animals.” Awareness of available treatment was a positive determinant of HIV testing. Blood transfusion as a perceived cause and asking advice from friends were negative determinants.

**Conclusions:**

Jordanian MSM seem to be aware of the risk of HIV infection and effective prevention methods, and they are willing to be tested for HIV. Our findings addressed the importance of the sexual meaning of HIV/AIDS on the control of HIV/AIDS among MSM. More effective engagement of NGOs and MSM in the prevention and control of HIV/AIDS is needed, enlisting the support of medical doctors and community health workers. Peer education should be strategically strengthened. Political commitment is needed to mitigate social stigma.

## Introduction

AIDS was first described as gay-related immune deficiency (GRID) syndrome, as it had initially been identified among men who have sex with men (MSM) in high-income countries. Despite huge efforts to control HIV/AIDS, rates among MSM in high-, middle- and low-income countries are increasing [1–3]. Data on HIV/AIDS in the Middle East and North Africa (MENA), especially for HIV infections among MSM, remain inadequate or unavailable [4, 5]. Despite a modest level of documented HIV prevalence, which was probably underestimated due to passive reporting limited by social, religious and political disincentives, the MENA region is currently one of two global regions where HIV incidence is increasing [6]. UNAIDS estimates more than half a million people are living with HIV/AIDS (PLWHA) in the region [6]. HIV prevalence is increasing among MSM [7], with concentrated epidemics in some countries of the region, namely Iran, Pakistan and Egypt [6, 7].

MSM may be the most hidden and stigmatized of all groups at risk of HIV infection in the MENA region. Stigma also complicates studying MSM in the Arab world [8]. MSM are vulnerable to homophobia resulting in harassment, discrimination and criminalization [9–11]. Information on characteristics and profiles of MSM populations in the MENA region is lacking for the above-mentioned reasons and their legal status, which varies from country to country. In Jordan, the Criminal Code allows adult, non-commercial and consensual homosexual relations above the age of consent, 16 years [12]. However, the rights of MSM are not legally protected in Jordan. No law or proposed legislation affords protection from discrimination or crimes based on sexual identity.

Control of HIV/AIDS in Jordan is implemented by the National AIDS programme (NAP) under the Ministry of Health. The NAP provides no-cost consultations, HIV testing and antiretroviral therapy through voluntary counselling and testing centres (VCTs) providing services only for HIV/AIDS. Due to political sensitivities, however, control of HIV/AIDS among MSM in Jordan and most of the MENA region is mainly implemented by local non-governmental organizations (NGOs) collaborating with the NAP. Programmes for MSM and other high-risk groups are new in the region. Key strategies to reduce HIV transmission among them rely on promoting HIV testing and condom use. Other strategies include raising awareness of HIV/AIDS, peer education and addressing the legal, psychological and social needs of MSM [13–15].

Condoms, if used correctly and consistently, prevent infection with HIV and other sexually transmitted diseases [16]. Condoms are widely available in the region for contraception. They are also offered by VCTs without charge. Awareness of one's HIV status through HIV testing is essential in preventing HIV transmission and promoting treatment of MSM [17]. Anonymous HIV testing at no cost is available from VCTs in Jordan and across the region.

Condom use and HIV testing, however, remain underutilized across the MENA region [6, 7]. Estimates for HIV testing are relatively low: 2–22% of MSM in Egypt, 22% in Lebanon [18] and 22% in Tunisia [7] have ever tested for HIV in 2010.

In Jordan, the recent Integrated Biological and Behavioural Survey (IBBS) in 2010 is the only available study of HIV/AIDS among MSM [19]. The IBBS found a laboratory-confirmed HIV prevalence of 0.2% in a sample of 468 MSM. In that sample, condoms were consistently used in the last 6 months by 19% for non-commercial and by 36% for commercial sexual intercourse. Condom use rates for last non-commercial and commercial sexual intercourse were 37 and 61%, respectively. Among MSM respondents, 32% had been previously tested for HIV.

The low condom use and HIV testing rates among MSM in the MENA region might be partly explained by Islamic values and cultural norms that may compromise the effectiveness of local HIV/AIDS control programmes. The recent increase in HIV infections suggests a need to study preventive behaviours of MSM and the impact of local sociocultural features of HIV/AIDS.

We aimed to identify determinants of self-reported condom use and HIV testing among MSM in Jordan. We also studied accessibility, motivation and obstacles for condom use and HIV testing. Among potential determinants, we considered HIV/AIDS-related knowledge, risk perception, perceived causes (PCs) and support available to MSM.

## Methods

### Setting

The study took place over a course of 5 months in 2011. Respondents were recruited from four main cities of Jordan (Amman, Aqaba, Irbid and Zarqa) through a partnership with an NGO called “Friends of PLWHA.” Although this NGO was established in 2009 with a public mandate to work with PLWHA, it also provides unpublicized consultations and supports high-risk groups, including MSM and female sex workers.

The study was approved by the Ethics Committee of Basel Region (Ethikkommission beider Basel, EKBB), Switzerland. After explaining the purpose and objectives of the study, participants provided verbal informed consent prior to being interviewed.

### Instrument

The study used a semi-structured explanatory model interview based on the framework and methods of cultural epidemiology [20, 21]. A semi-structured interview was constructed in accordance with the structure of the Explanatory Model Interview Catalogue (EMIC) [22]. EMIC interviews integrate quantitative and qualitative data to explain illness meaning, experience and behaviour in their local cultural context. Focus group discussions were conducted with representatives of the MSM community and with members of the NGO to inform development of the EMIC interview with reference to the context and study group. A first version of the interview was drafted in English, translated into Arabic and checked for validity and applicability in pilot interviews with five MSM; these interviews were not included in the analysis. The interviews were conducted by the first author and a trained representative from the NGO.

Participants were not asked directly about their HIV status to avoid discouraging their engagement. Questions of the EMIC interview referenced a clinical vignette depicting a person with typical somatic symptoms of AIDS. Respondents were asked to identify the condition. The interview considered locally relevant HIV/AIDS-related knowledge, risk perception, awareness of services and sources of support. The local meaning of HIV/AIDS was elicited by asking an open-ended question (“What do you think has caused the illness described in the vignette?”), followed by probing predefined categories of PCs related to injury, sexuality, religion, pathogens and illicit drugs. The interview also included questions on condom use during the respondent's last sexual intercourse and the respondent's history of HIV testing. Categorical data were elaborated with narratives that constituted a complementary qualitative component of the EMIC data set to help explain quantitative findings.

### Design and sampling

Among MSM known to the NGO, all potentially eligible men were approached by a member of their staff. Recognizing the high social stigma against MSM, the place and time of the interview were decided by each respondent individually. Categorical and narrative data were written down during the interviews. Interviews were also voice-recorded if respondents permitted it.

### Data management and analysis strategy

Quantitative data were entered twice, cleaned and verified in Epi Info 3.4.3. Statistical analysis was done using Stata 10. MAXQDA 2007 was used for managing and analysing textual data.

Sample characteristics, HIV/AIDS-related knowledge, risk perception, access to services and sources and obstacles for condom use and HIV testing were reported as frequencies. Categories of PCs were coded and analysed for their relative prominence (2=reported spontaneously; 1=reported only after probing the category; 0=not reported). An additional value of 3 was assigned if the category was considered the most important among all reported categories, so that each category received a value ranging from 0 to 5 for each respondent. This approach, based on prominence, distinguishes how categories were reported from consideration only of the frequency of reporting. The mean prominence for the sample of each PC category was calculated based on the prominence ranking for each respondent.

We considered two dichotomous outcome variables: condom use and HIV testing. The variable for condom use was coded with a value of 1 if respondents had used a condom at last intercourse and a value of 0 if they had not used a condom at last intercourse. HIV testing was coded with a value of 1 if respondents had ever been tested, and a value of 0 if they had never been tested for HIV.

For each outcome, we conducted a bivariate logit regression with each explanatory variable (i.e. HIV/AIDS-related knowledge, risk perception, support options and PCs). All variables with a *p*≤0.20 in the bivariate analysis were each independently retained for further logit regression adjusted for sample characteristics that are correlated with each outcome. Condom use determinants were independently adjusted for education level. HIV testing determinants were also independently adjusted for education level and occupation. We report adjusted logit regression coefficients with 95% confidence intervals.

## Results

### Sample characteristics

Out of 112 MSM who were contacted, 97 consented to be interviewed. Sample characteristics, stratified by reported condom use and HIV testing, are summarized in Table 1. The majority of respondents were single (93%), Muslims (92%) and students (56%). No one declared a positive HIV status. One-fourth reported condom use at last intercourse. Thirty-eight per cent had tested at least once for HIV. Only education was significantly associated with reported condom use at last intercourse. Occupation and education were associated with HIV testing.

**Table 1:** Sample characteristics by condom use and HIV testing among MSM in Jordan, *N*=97

	Total		Used condom last intercourse			Ever tested for HIV		
	
	*n*	%	*n*	%	*p*	*n*	%	*p*
Total	97	100.0	26	26.8		37	38.1	
Age category (years)								
17–25	73	75.3	21	21.6		27	27.8	
>25	24	24.7	5	5.2		10	10.3	
Marital status								
Single	90	92.8	24	24.7		33	34.0	
Ever married	7	7.2	2	2.1		4	4.1	
Religion								
Muslim	92	94.8	24	24.7		34	35.1	
Other	5	5.2	2	2.1		2	2.1	
Education								
Less than secondary	13	13.4	2	2.1	*	10	10.3	*
Secondary certificate	44	45.4	7	7.2	**	9	9.3	**
Diploma and higher	40	41.2	17	17.5		18	18.6	
Occupation								
Student	54	55.7	16	16.5		18	18.6	
Employed	32	33.0	6	6.2		13	13.4	
Unemployed	11	11.3	4	4.1		6	6.2	*

Unadjusted bivariate logit regression for sample characteristics categories with each outcome variable.

* ≤0.20, bivariate logit regression

** ≤0.05, bivariate logit regression.

### Condoms: access, sources and obstacles

In response to more general questions about condom use, 10% reported using condoms always, and the majority used condoms sometimes (67%) or never (23%). Eighty-four per cent spontaneously mentioned condoms as a means of HIV prevention, and 85% reported having easy access to condoms. Pharmacies were reported as a source for condoms by 88%; 52% reported friends as a source, and 21% obtained condoms from supermarkets. VCTs were the most infrequently reported source (9%).

Reduced pleasure was the most frequently reported reason for not using condoms (58%). Ineffectiveness of condoms was reported by 37%; in most cases, this meant rupture or slipping of condoms. Forty-one per cent stated they do not need condoms because they know their partners, or because they did not practice anal intercourse. Twenty-one per cent of MSM reported stigma as an obstacle, mainly because they felt ashamed to ask for or buy condoms. Among those who reported stigma as an obstacle, 90% were single and younger than 25 years. Only 4% reported cost as an obstacle to obtain condoms. Other reasons included religion and partner refusal; each of these was mentioned by less than 5%.

### HIV testing: access, motivation and obstacles

Sixty per cent knew that HIV testing was available in Jordan. The following testing facilities were mentioned: governmental health centres (43%), private health centres (32%), VCTs (11%) and NGOs (2%). Of those who had ever tested, 92% had done so voluntarily. The median number of tests was one test (range: 1–16 tests). Almost all tests (95%) were done after 2009. Testing was done by 41% out of self-motivation; recommendations by friends and by VCTs were reported by 32% and 16%, respectively. Of those who sought advice from VCT, 92% had tested for HIV.

“No need for the test” was the most frequent reason for not testing, reported by 52%. Respondents felt that they did not need the test mainly because they trusted their partners. Stigma was reported by 51% as an obstacle to testing. Qualitative data show that stigma mainly arose from interactions with healthcare workers, compounded by concerns about the confidentiality of testing. Because of stigma, some MSM stated that they donated blood to find out their HIV status and to bypass centres for HIV testing. Thirty-two per cent of respondents did not test since they were afraid of knowing their HIV status; 16% did not test since they were not sure about the confidentiality of test results. Costs and limited availability of tests as obstacles to testing were reported by 5 and 4%, respectively.

### HIV/AIDS-related knowledge, risk perception and support

All respondents had heard about HIV/AIDS, and 62% recognized it from the introductory clinical vignette (Table 2). Cancer, diabetes and ulcers were the most frequently identified diseases other than HIV/AIDS. Thirty-two per cent believed that treatment is available for HIV/AIDS, including energy therapy, self-help and painkillers; but only 12% mentioned antiretroviral treatment (ART).

**Table 2:** HIV/AIDS-related knowledge, risk perception and support by condom use and HIV testing among MSM in Jordan, *N*=97

	Total		Used condom last intercourse			Ever tested for HIV		
	
	*n*	%	*n*	%	*p*	*n*	%	*p*
HIV/AIDS-related knowledge								
Recognize HIV/AIDS from vignette	60	61.9	16	16.5		23	23.7	
Condoms can prevent HIV/AIDS	81	83.5	25	25.8	*	27	27.8	**
Aware of any available treatment	31	32.0	7	7.2		20	20.6	**
HIV/AIDS-related risk perception								
Personally afraid of infection	68	70.1	21	21.6		27	27.8	
Consider MSM a high-risk group	60	61.9	21	21.6	**	23	23.7	
HIV/AIDS is a problem in Jordan	53	54.6	18	18.6	*	22	22.7	
HIV/AIDS is a problem in the MENA region	68	70.1	21	21.6	*	27	27.8	
Support seeking								
Asked for advice regarding HIV/AIDS	60	61.9	20	20.6	*	24	24.7	
Sources for advice								
Friends	57	58.8	18	18.6	*	16	16.5	**
Internet	45	46.4	15	15.5		19	19.6	
Doctor	26	26.8	14	14.4	**	13	13.4	*
VCT†	25	25.8	9	9.3	*	23	23.7	
Teachers	13	13.4	6	6.2		6	6.2	

Unadjusted bivariate logit regression for HIV/AIDS-related knowledge, risk perception and support with each outcome variable.

VCT, voluntary counselling and testing centre; MENA, Middle East and North Africa.

† VCT was not included in the bivariate or multivariate testing analysis due to collinearity and high level of correlation

* *p*≤0.20, bivariate logit regression

** *p*≤0.05, bivariate logit regression.

Seventy per cent were personally afraid of an HIV infection, and 62% considered MSM as a high-risk group. A majority considered HIV/AIDS a problem in Jordan (55%) and the MENA region (70%). Major reasons were doubts about the official incidence and prevalence rates, inadequate control strategies, the taboo surrounding HIV/AIDS and risks arising from travel across borders of a foreign workforce (globalization).

Support (i.e. advice or help) regarding HIV/AIDS was sought by 62%, mainly to acquire information about disease prevention. Friends, including gay friends, were the most frequently mentioned source of advice (59%), followed by the internet (46%); doctors and VCTs were consulted by less than 27%.

Bivariate analysis selected the variables for inclusion in adjusted models (*p*<0.20) to explain condom use at last intercourse and previous HIV testing, as indicated in Table 2.

### Illness meaning

Unspecified sexual content was the most prominent PC, reported by 99% and identified as the most important cause by 78%. The variable “unspecified sexual content” was coded when participants referred to general sexual relations or sexual intercourse without specifying its nature. Homosexuality was identified by 2% as the most important PC. The HIV virus was reported as a cause by 69%, but mostly after probing. Some respondents explained that HIV is transmitted through pollution or through airborne or casual contact (e.g. shaking hands with an infected person).

Bivariate analysis of PCs associated with reported condom use at last intercourse and previous HIV testing for inclusion in an adjusted model (*p*≤0.20) are also indicated in Table 3.

**Table 3:** Perceived causes of HIV/AIDS by condom use and HIV testing among MSM in Jordan, *N*=97

	How reported (%)				Used condom last intercourse	Ever tested for HIV
	
Perceived causes^a^	Total^b^	Spon^c^	Important^d^	Mean^e^	*p*	*p*
Sexual content (unspecified)	99.0	93.8	78.4	4.28	*	**
Blood transfusion	91.8	87.6	12.4	2.16	*	**
Intravenous drug use	64.9	58.8	1.0	1.27		**
HIV virus	69.1	7.2	1.0	0.79		**
Homosexuality	45.4	7.2	2.1	0.59		
Injury or accident	48.5	2.1	0.0	0.51	*	
Sex with prostitutes	36.1	3.1	0.0	0.39	**	*
Pollution	20.6	5.2	2.1	0.32	*	
Sex with animals	29.9	1.0	0.0	0.31	*	*
Test from Allah	16.5	12.4	0.0	0.29		*
Fate or will of Allah	11.3	8.2	0.0	0.20		
Adultery	17.5	1.0	0.0	0.19	*	
Prior illness	14.4	1.0	0.0	0.15		*
Bad food or water	11.3	2.1	0.0	0.13		

Unadjusted bivariate logit regression for reported perceived causes of HIV/AIDS with each outcome variable.

a Perceived causes ordered by mean prominence, and causes reported by less than 10% not listed

b percentage of reported causes

c percentage of spontaneously mentioned causes

d percentage of causes identified as most important

e mean prominence based on values assigned to each reported perceived cause (0=not reported; 1=reported after probing; 2=reported spontaneously; and additional 3 if identified as most important)

* *p*≤0.20, bivariate logit regression

** *p*≤0.05, bivariate logit regression.

### Determinants of condom use

Adjusted analysis for education level revealed that acknowledging MSM as a high-risk group and seeking advice from a medical doctor were significantly positively correlated with condom use at last intercourse (*p*≤0.05) (Table 4). The PCs of sex with prostitutes and sex with animals were also significantly positively correlated.

**Table 4:** Determinants of condom use among MSM in Jordan, *N*=97

	Used condom last intercourse	
	
Variable	Adjusted coefficient†	95% CI
Consider MSM at high risk	1.28	(0.09–2.47)
Asked advice from doctor	1.95	(0.89–3.01)
Sex with prostitutes (PC)	1.56	(0.61–2.51)
Sex with animals (PC)	1.02	(0.05–1.99)

† Logit regression coefficient adjusted for education level; CI, confidence interval; PC, perceived cause.

### Determinants of HIV testing

Logit regression adjusted for education level and occupation revealed that awareness of available treatments and asking advice from a medical doctor were significantly positively correlated with HIV (Table 5). Blood transfusion and HIV virus as PCs, and asking advice from friends, were significantly negatively correlated with testing.

**Table 5:** Determinants of HIV testing among MSM in Jordan, *N*=97

	Ever tested for HIV	
	
Variable	Adjusted coefficient†	95% CI
Aware of any available treatment	1.46	(0.46 to 2.46)
Asked advice from friend	−1.55	(−2.88 to −0.23)
Asked advice from doctor	1.74	(0.29 to 3.19)
Blood transfusion (PC)	−1.05	(−1.80 to −0.30)
HIV virus (PC)	−0.99	(−1.81 to −0.19)

† Logit regression coefficient adjusted for education level and occupation; CI, confidence interval; VCT, voluntary counselling and testing centre; PC, perceived causes.

## Discussion

Decreasing HIV incidence rates and recent research indicating the use of ART as prophylaxis are reasons for optimism about the prospects for HIV/AIDS control on the global level [23–25]. Low prevalence rates and functioning health systems present a good opportunity to improve HIV/AIDS control in the MENA region. This is the first study to explore determinants of condom use and HIV testing among MSM in this region.

Condom use and HIV testing are two very important interventions for HIV/AIDS control. Our findings indicate low and inconsistent condom use among MSM, lower than the IBBS estimates in Jordan [19] and lower than reported numbers in Lebanon [18], Tunisia and Sudan [7], but higher than in Egypt [26]. The percentage of MSM who ever tested for HIV is higher than that reported in the IBBS study and higher compared to other regional estimates [7, 18].

Due to the sensitivity of the topic in the Arab world, MSM could not be randomly selected in this cross-sectional study in Jordan. This may limit the external validity of our findings since the recruitment through an NGO serving PLWHA may have biased the selection of MSM to a subset not fully representative of the target MSM population. We believe, however, that this approach to sampling through NGOs was appropriate under local circumstances and the only way to contact and talk to MSM. Making contact with MSM and encouraging them to talk openly about HIV/AIDS would not have been possible in a standard random sampling design. Moreover, the relatively small sample size limited our power to use multivariate analysis and further correction for confounding. Nevertheless, we believe the results shown here are of high importance and provide enlightenment about a highly understudied and stigmatized MSM population.

Global obstacles for condom use are accessibility, price, lack of awareness, reduced pleasure, social and cultural beliefs and so forth [27]. Accessibility, costs and lack of awareness were rarely reported in our study, whereas reduced pleasure, the perceived ineffectiveness of condoms and a lack of seeing a need for condoms were notable. Global and regional literature has reported similar obstacles to condom use among MSM populations, including issues related to (lack of) trust in partners [28–31]. An important finding from our study is that access-related stigma was an obstacle to condom use that was exclusively reported by young and unmarried MSM. Use of condoms as contraceptives is accepted in Jordan from a social and religious standpoint. This makes condom promotion for birth control possible, but it has been difficult to promote condom use for HIV control because it is socially unacceptable [32].

Promoting HIV testing indirectly increases awareness of HIV [33]. It is also well established that risky behaviours were reduced after testing [34, 35]. Meta-analytic evidence shows that most people who discover that they are HIV positive take preventive action, including condoms, to reduce the risk of transmission to others [34]. This is crucial in regions such as the MENA, where ambiguity still surrounds HIV/AIDS and many concerns regarding the actual burden persist.

Our findings showed good knowledge of HIV/AIDS, and awareness of condoms for prevention and availability of services (e.g. for HIV testing). Although risk perception was high, knowledge of available treatments and ART was low. Other studies from the region have also reported low awareness of ART within the general population and among PLWHA [36], but efforts to promote awareness and access to ART remain limited in Jordan and the wider MENA region [37].

Almost half of our respondents mentioned the internet as a source of advice. Past studies with MSM suggest that internet use rates for this group are higher than for other men [38, 39]. Other studies indicate that the internet has important implications for HIV/AIDS interventions among MSM [40, 41]. Internet-based methods may reach MSM who do not attend physical venues [42]. By June 2012, 40% of the population in the Middle East were frequent users of the internet; the world average at the same time was 34% [43]. These findings may likely be highly relevant for HIV/AIDS control among MSM in Jordan.

In our study, asking advice from friends was negatively associated with testing, probably due to questions of trust. Although many studies worldwide have found significant improvements in HIV testing rates and other healthcare services after peer-based interventions [29, 31, 44, 45], peer-to-peer education seems to be more complex in Jordan and possible the wider region. Gay friends may discourage MSM to test for HIV because they are afraid to be perceived as living with HIV or as not trustworthy. This finding does not suggest neglecting peer-to-peer education among MSM in Jordan; rather, it suggests rethinking different approaches to implement it, such as adequate training of peers who could encourage testing in their community. Peer-to-peer education among MSM may be a very potent tool for interventions, especially because of the high stigma towards this group.

Consideration of MSM being at high risk of HIV was positively associated with condom use in our study. A similar finding has recently been published about MSM in Beirut, where fear and anxiety emerged as motivators for both condom use and HIV testing [30]. Such a relation between risk perception and condom use or HIV testing has also been described in South Africa [28].

Although reported by only approximately one-quarter of respondents as a source of advice, getting information from medical doctors was a determinant of condom use and HIV testing. Medical doctors play an important role in prevention and diagnosis as they deliver reliable information. They should therefore be trained more in HIV/AIDS counselling for MSM. This may also help reduce the stigma that medical doctors and healthcare workers were found to attribute to HIV/AIDS in Jordan [46].

Stigma was also reported as a major obstacle to HIV testing in our study. HIV testing is stigmatized in the MENA region as it is connected to fear of infection that results in social isolation of MSM [47]. Stigma perceived by other gay men or the wider community and culture has been documented as a barrier to testing [33].

Whether ART was meant or not and regardless of the type or efficacy of treatment, awareness of available treatment was associated with HIV testing among MSM. Others also found a similar association, suggesting that awareness of available treatment may allay fear of positive test results [48]. However, awareness of treatment (i.e. ART) was also found to be associated with increased high-risk behaviours such as unprotected sexual intercourse [49]. Further research is needed to investigate the role of awareness of treatment on prevention in the region.

Nearly all MSM who sought advice from VCTs, the only places that offer free testing, were tested for HIV. It remains unclear, however, if visiting VCTs encouraged MSM to test, or if MSM preferred VCTs because of cost considerations or predisposition for testing. The former explanation seems more plausible because of two reasons: testing at VCTs has always been offered at no cost, and the majority of HIV tests in our sample were done after 2009 following a NAP policy change that supported more cooperation with MSM. Personal observation also suggests that HIV testing among MSM in general has increased in Jordan after this change. Further research needs to clarify the role of VCTs in the promotion of HIV testing in Jordan, and perhaps intensified efforts to encourage MSM to visit VCTs.

A belief that HIV tests are unnecessary was the most frequently reported reason against testing in our study. Testing for HIV seems to be more likely when individuals perceive themselves as being at risk of infection [50]. Being afraid of test results was also identified as an obstacle to testing, which has also been documented in the United States and Lebanon [30, 48]. However, the study from Lebanon showed that fear and anxiety also motivated some MSM to get tested.

The sociocultural features and meaning of HIV/AIDS among MSM may seem to play an important role in prevention and testing. MSM-reported preventive behaviours primarily acknowledged sexual transmission of HIV. Reporting sex-related PCs of the illness seem to be related to higher prevention as sex with prostitutes and sex with animals were positively associated with condom use. On the other hand, the respondent-perceived non-sexual meaning attributed to blood transfusion and the HIV virus was negatively associated with HIV testing. Interventions targeting MSM should take into account the meaning and their PCs of HIV/AIDS for more effective prevention and control in this particularly vulnerable subgroup of the population.

## Conclusions

MSM in our sample seem to be generally aware of the risk of HIV infection and effective prevention methods and are willing to be tested for HIV. While our findings indicate the importance of studying sociocultural factors to improve control of HIV/AIDS among MSM, some issues (e.g. the role of peer-to-peer education and VCTs for promotion of HIV testing) remain unclear and will benefit from further research. More effective engagement of NGOs in promoting HIV testing and prevention among MSM is needed. Medical doctors play an important role in control; reducing their discriminatory behaviour towards people with HIV and MSM could contribute to access and effectiveness of healthcare. More political commitment is also needed to mitigate social stigma of HIV/AIDS and the status of MSM, especially among healthcare workers.
